# Association between internal training load and muscle injuries in Brazilian professional soccer players

**DOI:** 10.5114/biolsport.2023.119285

**Published:** 2022-09-15

**Authors:** Pedro A Mohr, Thiago S Matias, Ricardo D de Lucas

**Affiliations:** 1 Federal University of Santa Catarina, Sports Center, Florianópolis, Brazil; 2 Avaí Futebol Clube, Florianópolis, Brazil

**Keywords:** Training load, Soccer, Rating of perceived exertion, Risk factor

## Abstract

The training load is associated with injury risk in a variety of sports. This study aimed to evaluate the association between the internal training load and injury risk in Brazilian professional soccer players. The data were collected from 32 soccer players across two full seasons (2017 and 2018). The rating of perceived exertion (RPE) for every training/match session was used as an internal load variable. The cumulative training load from 3 and 4 weeks (C3 and C4) and the acute:chronic workload ratio (ACWR) were calculated. A generalized estimating equation analysis was applied to examine associations of non-contact muscle injuries with C3, C4 and ACWR. A total of 33 injuries were recorded across the two full seasons. A significant association was found between cumulative training load for three (C3, p = 0.003) and four weeks (C4, p = 0.023) and the occurrence of injuries. Players in the “high load” group presented greater injury risk in relation to the “moderate load” group (C4: OR = 4.5; IC 95% 1.5–13.3; C3: OR = 3.7; IC 95% 1.7–8.1). There was no association between ACWR and injury occurrence. The athletes exposed to a high cumulative load in a period of 3 to 4 weeks presented higher injury risk in comparison to those who had moderate cumulative training loads. Besides that, there was no association between ACWR and injury occurrence.

## INTRODUCTION

Training load monitoring has become a common practice in sports, whereby the practitioners can assess whether an athlete is receiving a suitable training load [1]. It can be ascribed as internal or external workload, depending on the parameter assessed, as being internal or external to the athlete [2]. In the team sports context, there is increasing interest in the applicability of internal training load, which represents the perceptual and/or physiological responses of players from an external load. In soccer, the rating of perceived exertion (RPE) has been considered as a valid tool for measuring the internal load [3] and can be utilised for a variety of training sessions [4].

In the last few years, a topic of debate which has arisen is the possible relationship between training load and non-contact injury risk in different sports and athlete levels. Briefly, most of the studies have analysed this association by two different training load approaches. One consists of the association between the cumulated training load during a certain period of time (e.g., the absolute workload from one week, one month, etc) and the occurrence of injuries [5]. Another approach involves utilising the acute:chronic workload ratio (ACWR) (i.e., the magnitude of the current week’s training load in relation to a longer term training load, usually 3–6 weeks) [6]. Although it has been largely used in the field, it is noteworthy that ACWR has also been criticized because its causal relation to injury has not been established, lacking a conceptual basis and showing inconsistent results [7, 8].

According to the International Olympic Committee statement about load in sport and risk of injury, both acute and cumulated workload should receive special consideration [9]. Indeed, this association between training load and injury risk has been reported in elite soccer players from Europe [4, 8, 10] and Australia [11] by using RPE assessed following every training session.

Also, regional characteristics of soccer could influence the training/match demands and consequently the injury incidence [12]. For instance, lower injury incidence has been observed in professional Asian players than European players [13]. Regarding the relationship between workload and injury, even in the population of European players, there have been reported different results [4, 14]. To the best of our knowledge, there are no data reporting these relationships in professional Brazilian soccer players from a consistent period of time. Hence, the aim of the present study was to examine the association between absolute and relative internal training load and non-contact muscle injury risk in Brazilian professional soccer players.

## MATERIALS AND METHODS

### Participants

Data were collected from 32 professional soccer players from the same team (mean ± SD age: 27.5 ± 4.8 years, body mass 77 ± 8 kg, height 181 ± 7 cm) during two consecutive seasons (i.e., 2017 and 2018). Ten out of 32 players participated in both seasons, resulting in a total of 42 individual observations.

The team played in the Brazilian First Division Championship in 2017 and the Brazilian Second Division Championship in 2018. Only players who stayed in the club for at least 6 months took part in the analysis for the purpose of this research. The study was approved by the Human Research Ethics Committee of the local university, was performed in accordance with the ethical standards of the Helsinki Declaration, and participants signed an informed written consent form.

### Quantification of training load

The intensity of every session was quantified by the sRPE method [15]. The RPE score was collected approximately 30 minutes after every single training session (field and gym sessions) or match, always by the same staff member. Thus, the internal training load was derived from each player by multiplying the RPE score and the duration of training or match, and it was expressed as arbitrary units (AU). All data were gathered in a Microsoft Excel spreadsheet. Cumulative workloads were calculated considering blocks of 3 (C3) and 4 (C4) weeks (sum of daily training load, from Monday to Sunday), independently. To determine ACWR, the value of the last week (i.e., acute workload) was divided by the rolling average from the last 4 weeks (i.e., chronic workload) [1]. In the week of injury occurrence, the workload data from that week were not considered for calculations, regardless of the day of occurrence.

### Injury data collection

The occurrence of injuries was diagnosed by the team physician. An injury was considered when the player was unable to fully participate in future soccer training or a match due to any physical complaint [16]. Only acute (sudden-onset) non-contact muscle injuries were considered for analysis of the current study [17]. Muscle injury incidence was obtained by dividing the total number of muscle injuries by the exposure time (the sum of each session duration), and it was reported as the rate of injury per 1000 hours.

### Statistical analyses

Generalized estimating equations (GEE) were used to model the univariate association between load variables (ACWR, cumulated 3 and 4 weeks) and muscle injury occurrence in the subsequent week (i.e., the week of the injury was not included). The GEE analysis was chosen because it takes into account the correlated nature of the data [18]. To analyse the longitudinal data of a dichotomous response variable (i.e., injury: yes/no) the logit link function was used, with an exchangeable working correlation matrix [19]. When GEE analysis was significant (p < 0.05) the data were divided into tertiles, obtaining the following groups: “low load”, “moderate load” and “high load”. The “moderate load” group served as the reference for subsequent analysis. Odds ratios (OR) and 95% confidence intervals (CI) were calculated for comparison among injury risks in different load groups. All analyses were performed using the software SPSS v. 21.0 (IBM, USA).

## RESULTS

During the period of the study, a total of 501 training sessions and 124 matches were recorded. A total of 33 non-contact muscle injuries were detected over the two seasons (Season 2017, n = 14; Season 2018, n = 19). From the total, 17 occurred during matches and 16 during training sessions. The average incidence of non-contact muscle injury was 3.9/1000 h (2017, 3.6/1000 h; 2018, 4.2/1000 h).

The average cumulated training load during 4- and 3-week blocks was 4930 ± 1289 AU and 3672 ± 971 AU, respectively. There were significant associations of C4 and C3 with injury risk (C4: p = 0.023; C3: p = 0.003) (Table 1).

**TABLE 1:** Odds ratio (OR), 95% confidence interval (CI95%) and p-value between the cumulative training load, and binary outcome (injury: yes/no).

Cumulative workload	OR (IC 95%)	p-value
4 Weeks Workload > 5335	4.5 (1.5–13.3)	0.006*
4 Weeks Workload < 4260	2.5 (0.95–6.6)	0.062
3 Weeks Workload > 3980	3.7 (1.7–8.1)	< 0.001*
3 Weeks Workload < 3150	2.1 (0.8–5.4)	0.135

The reference workload range for the 4-weeks load: 4260 to 5335 AU; and for the 3-weeks load: 3150 to 3980 AU.

* Significant effect (p < 0.05)

**FIG. 1:**
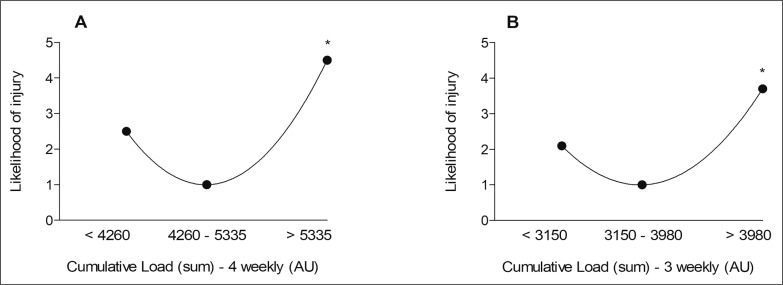
Cumulated workload and likelihood of injury for 4 weeks (Panel A) and 3 weeks (Panel B). * Likelihood of injury significantly greater than the reference group (p<0.05).

For both variables, the “high load” group showed greater injury risk than the “moderate load” group (C4: OR = 4.5; CI 95% 1.5–13.3; C3: OR = 3.7; CI 95% 1.7–8.1) (Figure 1). There was no difference in the injury risk between the “low load” group and “moderate load” group (p > 0.05).

**FIG. 2:**
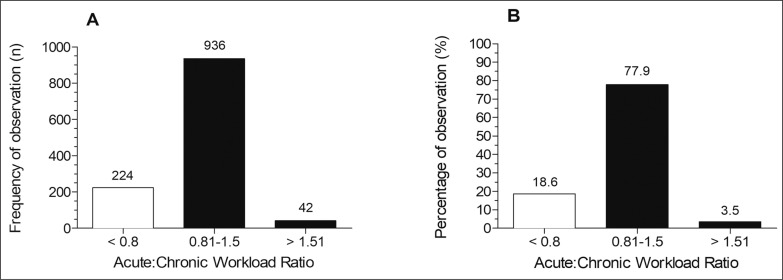
Frequency (Panel A) e percentage of observations (Panel B) according to acute:chronic workload ratio.

No significant association was found between ACWR and injury occurrence (p = 0.569). Figure 2 depicts the frequency and percentage of observations according to the reference zones as suggested elsewhere^1^.

## DISCUSSION

The present study aimed to analyse the relationship between internal training load and the risk of acute non-contact muscle injuries in a group of Brazilian professional soccer players. We found an association between accumulated training load and injury occurrence, for the two entire seasons analysed herein. Specifically, the high-cumulated workload during 4 and 3 weeks raised the injury risk in relation to the moderate cumulated workload, for the same period.

According to our results, the high-cumulated workload (above 5335 and 3980 AU for 4 and 3 weeks, respectively) raises the injury risk by 4.5 and 3.7 times compared to the moderate workload group. This U-shaped relationship between training load and injury, as observed in Figure 1, has been previously reported in the literature [20]. In soccer players, a decreased injury risk was found for moderate values of external load [21, 22]. Regarding the internal training load, it was also found that a high-cumulated training load was associated with a higher injury risk than the moderate training load [23]. It is well understood that augmented load is an important factor for improving physical fitness [24]. Indeed, cardiovascular and neuromuscular adaptations are triggered through a high training load, acquired by the manipulation of training intensity, volume and frequency [24, 25]. However, if the intensity or volume is above the capacity of the psychophysiological system to endure, supposedly it could produce a likely overload, triggering a subsequent soft tissue injury.

In relation to the low-cumulated workload, despite the tendency for an increased injury risk in this condition (Figure 1), a significant association was not found. It has been reported in female soccer players that a low average weekly exposure was associated with a higher risk of injury [26]. In other sports, this relationship has also been established [27, 28], which could be associated with under-training. Therefore, it is important to recognise the “ideal” workload zone, in order that the psychophysiological adaptations can occur without high exposure to injury occurrence [29].

Contrary to the present results, Malone et al. [10] found a linear association between injury risk and training load in soccer players from European teams. During the competitive period, the group with higher cumulated training load showed a lower injury risk than the group with low cumulated load. However, the 95% confidence interval of the OR included the value 1 (OR = 0.91; IC 95% 0.26–3.14), so the relationship is not reliable. Other studies have not found an association between cumulated training load and injury risk [4, 30]. Features such as competitive level, number of seasons analysed and the number of injuries can explain these disagreements. Also, the statistical model approach and the way the data categorization is arranged can challenge the comparison among studies [31].

The average weekly training load observed in the present study was slightly lower than previously reported in the literature [8, 23, 32]. A possible explanation is the massive number of games played in a continental-size country such as Brazil. In this way, the logistics of travelling have an impact on the number of training sessions, which could at least partly explain the lower weekly training load.

The use of ACWR for controlling the training load has spread in the last few years [9]. Its use is intended to identify the injury risk, consequently reducing the likelihood of a player presenting a soft tissue injury, which should be under the control of staff [33]. However, the ACWR has been criticised regarding the lack of a conceptual basis and inconsistent results [7, 8]. The ACWR is an extension of Banister’s fatigue-fitness model used to predict performance (which has been criticised for the lack of a physiological underpinning), without a clear link to mechanisms of injuries. Also, its relationship with risk of injuries often could appear in different directions [7]. In the present study, the ACWR was not significantly associated with the injury incidence, agreeing with part of the literature [14, 30]. While some authors have found an association between the ACWR and injury risk [4, 8, 21, 23], others did not observe a significant relationship [14, 30]. According to Gabbett [1], values above 1.5 represent a “danger zone” for the occurrence of a non-contact injury. In the present study, only 3.5% of injuries occurred at an ACWR above 1.5. Thus, based on the low occurrence of injuries within this zone, one could suggest that this metric is not sufficient to detect greater risk.

Regarding the studies in the literature about the relationship between training load and injury risk, it is important to mention that there has not been a distinction among injury types (e.g., an injury with an acute or repetitive mechanism). However, it is unlikely that the load-injury relationship is the same regardless of the nature of the injury [34]. Therefore, defining clearly which type of injury is being discussed can help in the understanding of this relationship.

Beyond injury risk management, a well-designed training programme should positively impact performance [35]. For example, a negative association was observed between low season injury rates and team performance (i.e., points won per league match) [36], Thus, the training load monitoring could assist in obtaining the most effective training “dose” for maximising the improvement of physical fitness (i.e., athlete’s resilience), while minimising injury risk, with a resultant increased player availability.

Based on the results of the present study, the monitoring of internal training load could be utilised for identifying soccer players with increased risk of muscle injury. The present data suggest that a high 3- and 4-week cumulative training load increases the muscle injury risk by at least 3 times. By using cumulative training load data, coaches could take better decisions in daily practice in order to increase player availability for training and competitions.

The fact that subjects belonged to the same soccer team should be considered as a strength of the current study, although extrapolation of the results should be done with caution. In addition, it is important to highlight that the team’s staff used the workload data to eventually adapt the training programming, and it may have impacted the outcomes. On the other hand, the present study could be considered as a ‘real world’ observation, with respect to the relationship among training prescription, training load and injury incidence in a high-level soccer team. As stressed elsewhere, the risk (and subsequent injury) management in elite sport should be supported by robust longitudinal data, such as the current study has presented [37]. Additionally, the injury definition utilised herein may differ from others studies, making comparison among them difficult. Finally, the complex system approach is rising in popularity, and future studies should take into account the multifactorial nature of sports injuries [38].

## CONCLUSIONS

In conclusion, cumulative training loads are associated with increased risk of acute non-contact muscle injury in professional Brazilian soccer players. Soccer players exposed to greater internal training loads in a period of 3 or 4 weeks are more susceptible to suffer from muscle injuries, while ACWR presented no significant association with injury risk. Thus, one should highlight the importance of monitoring the internal training load, in order to reduce injury risk in professional soccer players. Additionally, this reduction in injury risk can also help achieve a better performance.
